# Chronic cerebral hypoperfusion induces post-stroke dementia following acute ischemic stroke in rats

**DOI:** 10.1186/s12974-017-0992-5

**Published:** 2017-11-09

**Authors:** Dong Bin Back, Kyoung Ja Kwon, Dong-Hee Choi, Chan Young Shin, Jongmin Lee, Seol-Heui Han, Hahn Young Kim

**Affiliations:** 1 Department of Neurology, Konkuk University School of Medicine, Research Institute of Medical Science, Seoul, Republic of Korea; 2 Department of Medicine, Konkuk University School of Medicine, Seoul, Republic of Korea; 3 Department of Pharmacology, Konkuk University School of Medicine, Seoul, Republic of Korea; 4 Department of Rehabilitation Medicine, Konkuk University School of Medicine, Seoul, Republic of Korea; 5 Konkuk University Medical Center, 120-1 Neungdong-ro, Gwangjin-gu, Seoul, 05030 Republic of Korea

**Keywords:** Post-stroke dementia, Chronic cerebral hypoperfusion, Neuroinflammation, Glymphatic pathway, Amyloid pathology, Animal model

## Abstract

**Background:**

Post-stroke dementia (PSD) is one of the major consequences after stroke. Chronic cerebral hypoperfusion (CCH) can induce vascular cognitive impairment and potentiate amyloid pathology. We investigated how CCH contributes to the development of PSD after stroke in the context of neuroinflammation and amyloid pathology.

**Methods:**

We designed a unique animal model for PSD. We performed middle cerebral artery occlusion (MCAO) surgery in rats mimicking acute territorial infarct, which was followed by bilateral common carotid artery occlusion (BCCAo) surgery mimicking CCH. We performed behavioral tests including neurologic function test and water maze task and histological investigations including neuroinflammation, neuronal cell death, amyloid pathology, and aquaporin 4 (AQP4) distribution.

**Results:**

Spatial memory was synergistically impaired when BCCAo was superimposed on MCAO. Neuroinflammation with astroglial or microglial activation and amyloid pathology were enhanced in the ipsilateral cortex, thalamus, and hippocampus when BCCAo was superimposed on MCAO. Glymphatic pathway-related AQP4 distribution changed from perivascular to parenchymal pattern.

**Conclusions:**

Our experimental results suggest that CCH may contribute to the development of PSD by interfering with amyloid clearance through the glymphatic pathway and concomitant neuroinflammation. Therapeutic strategy to clear brain metabolic waste through the glymphatic pathway may be a promising approach to prevent PSD after stroke.

## Background

Stroke is the third leading cause of death following cancer and cardiovascular disease in most of the developed countries [1]. More than half of stroke survivors experience residual physical disability or cognitive decline [2]. Even in physically independent survivors, cognitive decline can be a major hurdle in returning to the premorbid social life. A quarter of stroke patients develop dementia within 3 months post stroke [3]. Any dementia developed or aggravated after stroke can be considered as post-stroke dementia (PSD) by the definition “post-stroke”. Although this concept is simple and useful to define PSD, its clinical manifestations are diverse from unmasking of a pre-stroke cognitive impairment to the newly developed dementia resulting from recurrent multiple infarcts or a single strategic infarct [4].

The pathophysiology of dementia is complicated and heterogeneous including vascular dementia (VD), Alzheimer’s disease (AD), or mixed dementia [5]. Considering the nature of PSD developing after stroke, PSD may be more related to VD including strategic infarct dementia, multi-infarct dementia, subcortical ischemic vascular dementia, hypoperfusion dementia, or mixed dementia rather than pure neurodegenerative dementia such as AD [4]. Single or recurrent cortical infarcts can lead to strategic infarct dementia or multi-infarct dementia [6], whereas subcortical infarcts such as lacunes or white matter lesions can be associated with subcortical ischemic vascular dementia or hypoperfusion dementia [6]. Pathology related to AD such as amyloid deposits can be observed in ischemic lesions or surrounding penumbra in animal experiments [7–10] or patients with stroke [11]. More complex interaction among white matter lesions, hypertensive angiopathy, and amyloid deposit in the brain parenchyma and vessels may contribute to the development of PSD rather than direct effect of stroke lesions [12]. Considering the complex interaction of AD and vascular pathology [13], pathophysiology of PSD may be complicated by the interaction between ischemic lesions and amyloid deposit.

Mechanism-mediating amyloid clearance have been reported, including enzymatic degradation, receptor-mediated blood-brain barrier clearance, cerebrospinal fluid (CSF) absorption clearance, and interstitial fluid (ISF) bulk flow clearance [14]. Recently, glymphatic pathway has drawn attention as an ISF bulk flow clearance for brain metabolic wastes such as amyloid, tau, and synuclein [14–17]. In addition, impaired clearance is associated with the development of neurodegenerative disease such as AD [14–17]. Considering that AD-related pathology was reported in the brain of patients with PSD [11], glymphatic pathway may provide a possible link among VD, AD, and mixed dementia comprising a diverse nature of PSD.

Previously, we showed that cognitive impairments in rats with permanent occlusion of bilateral common carotid arteries (BCCAo) suggest its usefulness as an animal model for VD induced by chronic cerebral hypoperfusion (CCH) [18–20]. Cognitive impairments in this animal model were associated with white matter disintegration [18, 20]. Considering that white matter lesions induced by CCH are one of the predisposing factors for PSD, we hypothesized that CCH may play a key role in the development of PSD after stroke.

To simulate interaction between stroke and underlying CCH in the development of PSD, we designed a unique animal model for PSD. We performed transient occlusion of the middle cerebral artery (MCAO) for 90 min in rats mimicking a territorial infarct, which was followed by BCCAo mimicking a CCH. Using this model, we investigated how CCH contributes to the development of PSD after stroke in the context of neuroinflammation and amyloid pathology.

## Methods

### Experimental design

**Fig. 1:**
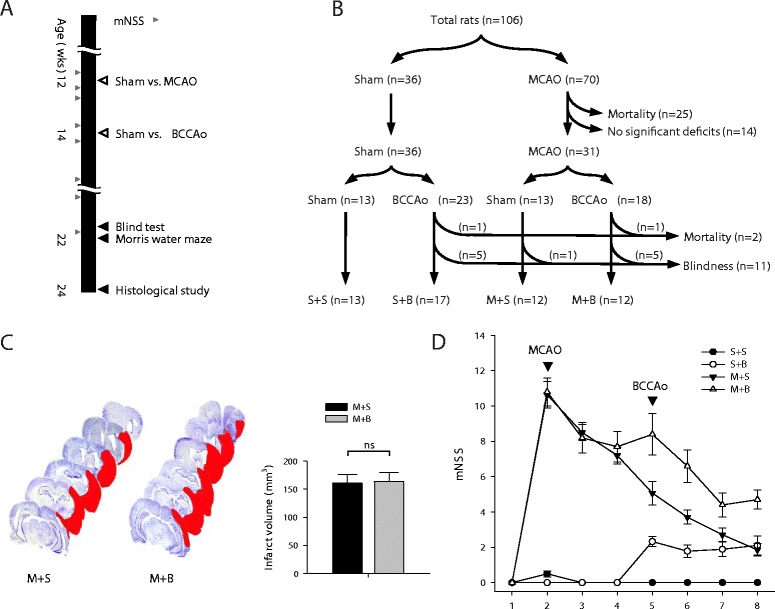
**a** The timeline of the experiment. **b** Allocation of rats into groups. **c** Representative images of cresyl violet staining and quantification of infarct volume in M + S and M + B groups. *n* = 10 in both groups. **d** Serial neurologic function test. *n* = 12 to 17 in each group. MCAO middle cerebral artery occlusion, BCCAo bilateral common carotid artery occlusion, S + S sham + sham, S + B sham + BCCAo, M + S MCAO + sham, M + B MCAO + BCCAo, mNSS modified neurologic severity scores, ns not significant

Male Wistar rats were used in these experiments (aged 3 months; 300–350 g; Orient Bio, Seoul, Republic of Korea). All rats resided in the vivarium at Konkuk University for 2 weeks before the beginning of the experiment. The rats were housed at a controlled temperature (22 ± 1 °C) and humidity (50 ± 10%) on a 12-h alternate light-dark cycle. Food and water were provided ad libitum throughout the experiment. All animal experimental procedures were performed in accordance with the approved guidelines of the Institutional Animal Care and Use Committee of Konkuk University (Approval number KU16070) and ARRIVE guidelines (https://www.nc3rs.org.uk/arrive-guidelines). Sequential surgeries, i.e., initial MCAO surgery followed by BCCAo surgery after 2 weeks, were performed (Fig. 1a). The rats were divided into four groups as follows: MCAO followed by BCCAo (M + B) or sham operation of BCCAo (M + S) and sham operation of MCAO followed by BCCAo (S + B) or sham operation of BCCAo (S + S). Based on the calculation using previous data of mortality and blindness after surgery [18, 20], the rats were allocated randomly to sham operation or MCAO at a ratio of 1:2 (Fig. 1b). Transient MCAO was achieved with a standard intraluminal suture approach for 90 min as described elsewhere [21]. The BCCAo was performed as described elsewhere [18–20]. Briefly, the bilateral common carotid arteries were carefully exposed through a midline incision and were permanently double-ligated with silk sutures. As time went on after the BCCAo, cerebral blood flow decreased chronically down to 70% of the baseline in our previous study [20]. Rats were anesthetized using a 5% isoflurane/oxygen mix and maintained on 3% isoflurane/oxygen during the surgery. The rectal temperature was maintained between 37 ± 0.5 °C with a heating pad during the surgery. The timeline of the experiment is shown in Fig. 1a. Motor function recovery was serially measured by modified neurologic severity scores (mNSS). After excluding rats with impaired vision by blind test, Morris water maze task was performed for 2 weeks, starting from 10 weeks after the initial surgery. For histological evaluation, rats were sacrificed after all behavioral experiments were performed. Rats were coded with numbers, and all investigators were blinded to the treatment groups until the end of the data analysis.

### Neurologic function test

The mNSS, ranging from 0 to 14, is a composite scoring system for the neurologic function composed of motor status, abnormal movements, sensory tests, and reflexes [22]. For each individual task, one point is awarded for the inability to correctly perform. The higher the score is, the more severe neurological injury it suggests.

### Blind test

Prior to the water maze task, blind test was performed as previously described [20]. Rats were placed in a 90 cm × 45 cm × 32 cm (height) sized box separated by a wall with an open path in the middle (18 cm × 32 cm). The floor of one space was gridded with electrodes and that of another space was flat. After free exploration of the space for 5 min, rats were put into the grid space and given a foot shock with 3 mA for 1 s. Under daylight, rats with normal vision could escape to a safe space through the open path immediately after the shock, while blinded rats were unable to find the path and turned around repeatedly in the grid space. Escape latency longer than the mean plus three standard deviations of sham-operated rats was used as an arbitrary cutoff to determine blindness.

### Water maze task

Rats were evaluated in a Morris water maze as previously described [18–20]. The maze was a round tank with 1.83 m of diameter and 0.58 m deep, filled to a depth of 35.5 cm with tepid (26 ± 1 °C) water made opaque by the addition of white paint. A moveable circular platform with 12 cm of diameter was located 2 cm below the surface of water. The maze was surrounded by white curtains on which black visual stimuli of various shapes and sizes were placed. A camera which relayed images to a videocassette recorder and an HVS Image Analysis Computer System (Hampton, UK) was located above the center of the maze. Four consecutive sessions, each consisting of five trials for 2 days (alternating two or three trials per day), were conducted on eight consecutive days. The hidden platform was constantly located in the southeast quadrant of the pool. Rats were gently submerged in water, facing the inside wall of the tank. Every trial started at different points, alternating among four quadrants. Rats were gently handled for 10 min daily for 7 days before the test. In the maze, the rats were allowed to swim for a maximum of 90 s. Further, they were allowed to remain on the platform for 30 s at the end of each trial. Performance accuracy was evaluated by the analysis of search error, time latency, and path length data of all trials. Measurement of the search error was based on a computation of the average distance from the platform during the trial. The distance between the rat and the platform was sampled 10 times/s during each trial, and these distances were averaged in 1-s bins. Cumulative search error is the sum of these 1-s averages of the proximity measure corrected for the specific platform location and start location by subtracting the proximity score that would be produced by a perfect performance in the trial. A probe trial was conducted 1 min after every 10th training trial. Therefore, rats took the second probe trial after finishing 10 more training trials over 4 days after the first probe trial. The entire training procedure included two probe trials for each rat, during which the rats swam with the platform retracted to the bottom of the pool for 30 s. After recording the swimming path, the platform was raised to its normal position for completion of the trial. The swimming time spent in the target quadrant and the number of crossings above the retracted platform were used as parameters for the retention of spatial memory.

### Tissue preparation

After all behavioral experiments, the rats were intracardially perfused with 0.01 M phosphate-buffered saline (PBS) and subsequently with 4% paraformaldehyde (PFA) under deep anesthesia. The brain was removed quickly and post-fixed in 4% PFA for 2 days, cryoprotected in PBS containing 30% sucrose for 48 h, frozen in powdered dry ice, and sectioned using a microtome at a thickness of 30 μm.

### Cresyl violet staining

Sections were mounted onto resin-coated slide glass, dried for 10 days, hydrated through descending concentrations of ethanol, and subsequently dipped twice in distilled water. Sections were immersed in cresyl violet acetate (Sigma Aldrich) dissolved at 0.5% (*w*/*v*) in distilled water for 5 min and then dehydrated through ascending concentrations of ethanol. Finally, they were defatted in xylene and coverslipped with permount reagent.

### Immunohistochemistry

The sections were washed in PBS with 0.3% Triton X-100 and then incubated in blocking serum, 5% normal donkey serum in 0.15% triton with PBS. Subsequently, they were incubated in primary antibody solution for 1 h at room temperature and overnight at 4 °C as follows: mouse anti-glial fibrillary acidic protein (GFAP) (1:1000, BD Bioscience), rabbit anti-ionized calcium-binding adapter molecule-1 (Iba-1) (1:1000, Wako), rabbit anti-NeuN (1:1000, EMD Millipore), and mouse anti-amyloid beta_17-24_ (4G8) (1:100, Covance), rabbit anti-collagen IV (1:1000, Abcam), and mouse anti-aquaporin 4 (AQP4) (1:100, Abcam). Then, the sections were washed in PBS with 0.15% Triton X-100 and incubated in a secondary antibody solution for 3 h at room temperature as follows: anti-mouse Alexa Fluor 488 and 568; anti-rabbit Alexa Fluor 488 and 568; all from donkey (1:200, Invitrogen). Stained sections were mounted on resin-coated slides and dried for 30 min. Slides were then coverslipped with ProLong® Gold antifade reagent (Invitrogen).

The histological staining of amyloid beta was confirmed by the approach of 3, 3′-diaminobenzidine (DAB) staining. The sections were incubated with 3% hydroxide and 3% methyl alcohol for quenching endogenous peroxidase activity. They were then blocked and incubated with 4G8 (1:100, Covance) overnight at 4 °C. The sections were subsequently washed and incubated in a secondary antibody solution (biotinylated horse anti-mouse antibody, Vector, 1:200) for 1 h at room temperature. Finally, peroxidase activity was visualized using DAB solution (Vector SG kit, Vector).

### Quantitative analysis

Signal intensity of GFAP and Iba-1 immunoreactivity was evaluated from the section at bregma 0.20 mm for the cortex and striatum and at bregma − 3.30 mm for the thalamus and hippocampus. Images encompassing a region of interest (ROI) were captured using a confocal microscope (Olympus FV1000). Ipsilateral ROIs in the cortex and striatum of M + S and M + B rats were selected in the peri-infarct area. Contralateral ROIs were selected as symmetrical regions in the contralateral hemisphere. The ROIs were pictured with a 20X/0.75 NA objective in 1024 × 1024 pixels (field size 653.283 × 653.283 μm^2^ for the cortex, striatum, and thalamus) and a 4X/0.16 NA in 1024 × 1024 pixels (field size 3178.461 × 3178.461 μm^2^ for the hippocampus). Fluorescence signal intensity in the ROIs was evaluated using the FluoView v.3.1 software (Olympus) and expressed as the relative percentage change compared to those of S + S rats.

### Amyloid deposit

Number and area of amyloid plaques in the cortex were summed from sections at bregma 0.20 and − 3.30 mm. An amyloid plaque was defined as a conglomeration comprised of degenerated neurons surrounded by amyloid fibrils stained by 4G8. Single degenerated neuron occupying less than 10 μm^2^ was not considered as an amyloid plaque. The number of amyloid plaques was manually counted, and the area of each amyloid plaque was manually drawn and calculated using NIH Image J (Bethesda, MD, USA).

### Distribution of AQP4

Distribution patterns of AQP4 immunoreactivity were analyzed. The AQP4 signals colocalized with collagen IV were considered as vascular AQP4 and those not-colocalized with collagen IV were considered as parenchymal AQP4. An arbitrary low stringency threshold was set to define the total area of AQP4 immunoreactivity, whereas a high stringency threshold was set to define an area of vascular AQP4 which was colocalized with collagen IV. The ratio between the areas of vascular and parenchymal AQP4 was compared among groups. Moreover, the area ratio of vascular AQP4 to collagen IV was defined as AQP4/Col IV colocalization index, reflecting perivascular astrocytic end feet coverage of microvessels. Area calculations were performed using images converted into black and white by an equal threshold to all individual images using NIH Image J (Bethesda, MD, USA).

### Infarct volume

The infarct volume was calculated using an indirect method based on six representative cresyl violet-stained slices selected with 2-mm interval.

### Hippocampus

The number of viable or degenerative neurons in the hippocampus was evaluated by cresyl violet or 4G8, respectively. Rectangular ROIs occupying 100 × 100 μm^2^ were selected in the CA1 and CA3 regions selected from section at bregma − 3.30 mm. The neurons were counted using a cell counter available in NIH Image J (Bethesda, MD, USA) and expressed as the number of cells per mm^2^.

### Statistical analysis

Parameters for spatial memory including search error, time latency, path length, and swimming speed were analyzed by one-way repeated-measure ANOVA followed by a post hoc Tukey’s honest significant difference test. One-way ANOVA was conducted to compare results of the probe trials and the quantitative data of the immunohistochemistry (mean ± SE). A value of *p* < 0.05 was considered to be statistically significant. Data analyses were performed with the SPSS Statistics 24.0.

## Results

### Experimental groups

One-hundred and six rats were distributed into four groups with sizes ranging from 12 to 17 per group (Fig. 1b). Mortality rate for MCAO and BCCAo surgeries was 35.7% (25 of 70) and 4.9% (2 of 41), respectively. Rats without significant neurological deficits (mNSS 0 to 2) after MCAO surgery (*n* = 14) were excluded due to the possibility of no infarct. Rats that failed to pass blind test were excluded from the water maze task, which depends on intact visual function (*n* = 11).

### Infarct volume and neurologic function

Although infarct volume was slightly larger in M + B rats than in M + S rats, it was not statistically significant (*p* = 0.895, Fig. 1c). Additional BCCAo surgery did not increase the infarct volume of MCAO surgery. The neurologic functions of the rats deteriorated immediately after MCAO or BCCAo surgery. However, functions recovered enough to perform the water maze task (Fig. 1d).

### Spatial memory

**Fig. 2:**
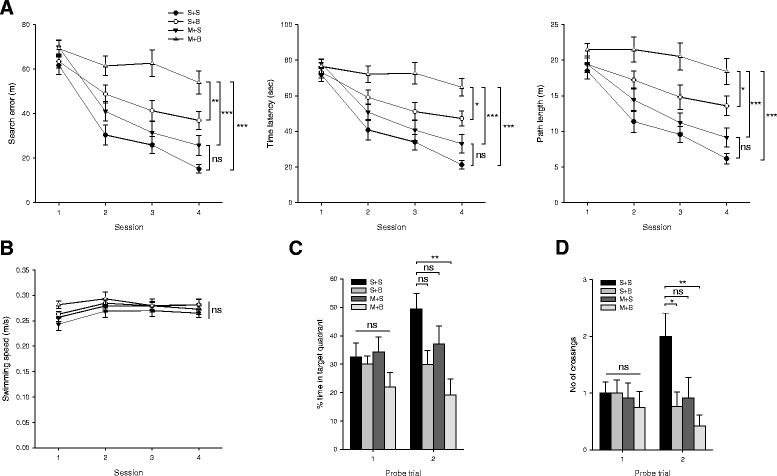
Morris water maze task. **a** Search error, time latency, and path length. **b** Swimming speed. **c** Time staying in the target quadrant during probe trials. **d** Number of crossings over the removed platform during probe trials. *n* = 12 to 17 in each group; *p < 0.05, **p < 0.01, and ***p < 0.001 on post hoc analysis; MCAO middle cerebral artery occlusion, BCCAo bilateral common carotid artery occlusion, S + S sham + sham, S + B sham + BCCAo, M + S MCAO + sham, M + B MCAO + BCCAo, ns not significant

Four experimental groups showed statistically significant difference in the performance of the water maze task (*p* < 0.001 in search error, time latency, and path length, Fig. 2a). Post hoc analysis revealed that M + B rats showed poorer performance in the search error than did S + S and M + S rats (*p* < 0.001) or S + B rats (*p* = 0.007). However, the search error was not different between S + S and M + S rats (*p* = 0.216). Other parameters including time latency and path length showed similar pattern. Swimming speed was not different among groups (*p* = 0.377, Fig. 2b). In the first probe trial, the time in the target quadrant and the number of crossings were not different among groups (*p* = 0.253 and *p* = 0.876, respectively; Fig. 2c, d). However, in the second probe trial, the time in the target quadrant and the number of crossings were significantly different among groups (*p* = 0.004 and *p* = 0.006, respectively; Fig. 2c, d) suggesting most effective learning in S + S rats.

### Neuroinflammation

**Fig. 3:**
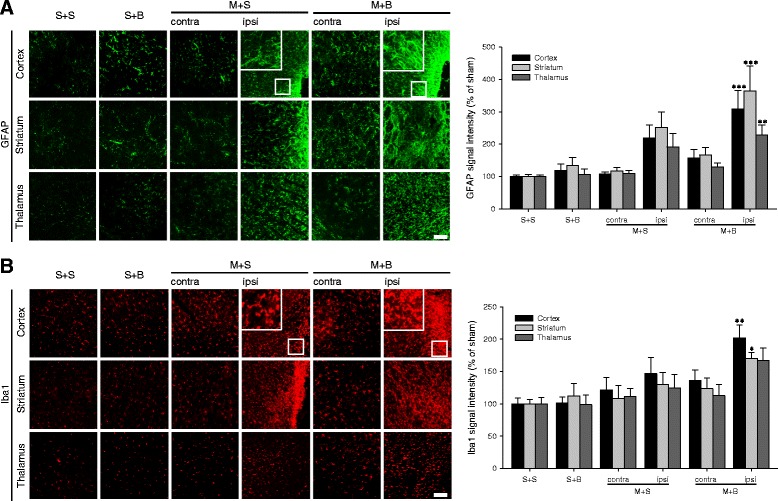
Neuroinflammation measured by astroglial and microglial activation. **a** Glial fibrillary acidic protein (GFAP) immunoreactivity and its quantification. **b** Iba1 immunoreactivity and its quantification. *n* = 6 to 8 in each group; scale bar = 100 μm; *p < 0.05, **p < 0.01, and ***p < 0.001 on the post hoc analysis compared to S + S; MCAO middle cerebral artery occlusion, BCCAo bilateral common carotid artery occlusion, S + S sham + sham, S + B sham + BCCAo, M + S MCAO + sham, M + B MCAO + BCCAo

Astroglial activation localized with GFAP increased in the ipsilateral cortex of M + B rats followed by the ipsilateral cortex of M + S rats and the contralateral cortex of M + B rats (*p* < 0.001, Fig. 3a). Post hoc analysis revealed that the astroglial activation significantly increased in the ipsilateral cortex of M + B rats compared to that in S + S rats (*p* < 0.001). Microglial activation localized with Iba-1 increased in the ipsilateral cortex of M + B rats followed by the ipsilateral cortex of M + S rats and the contralateral cortex of M + B rats (*p* = 0.001, Fig. 3b). Compared with S + S rats, M + B rats showed a significant increase in microglial activation in the ipsilateral cortex (*p* = 0.001). Both neuroinflammatory markers showed similar patterns in the striatum and in the thalamus as those in the cortex.

### Amyloid deposit

**Fig. 4:**
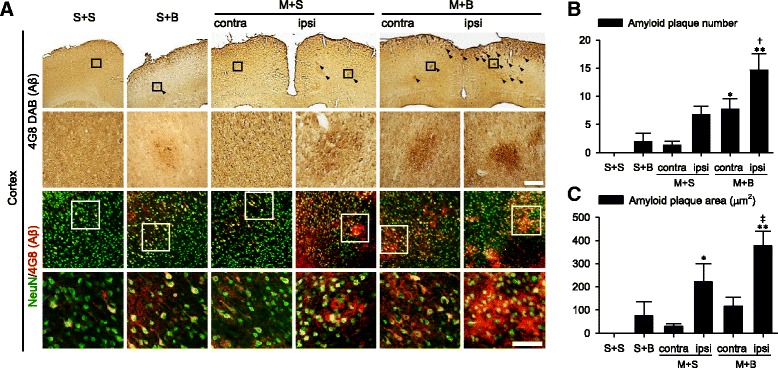
Amyloid deposit in the cortex. **a** Amyloid plaques are indicated in 4G8 DAB staining (arrow heads) and double immunofluorescent labeled by neuron (NeuN) and amyloid deposit (4G8). **b** Quantification of amyloid plaque number and **c** area. *n* = 6 to 8 in each group; scale bar = 100 μm; *p < 0.05, **p < 0.001 on the post hoc analysis compared to S + S; †*p* < 0.05 on the post hoc analysis compared to ipsilateral M + S; ‡*p* < 0.05 on the post hoc analysis compared to contralateral M + B; MCAO middle cerebral artery occlusion, BCCAo bilateral common carotid artery occlusion, S + S sham + sham, S + B sham + BCCAo, M + S MCAO + sham, M + B MCAO + BCCAo

**Fig. 5:**
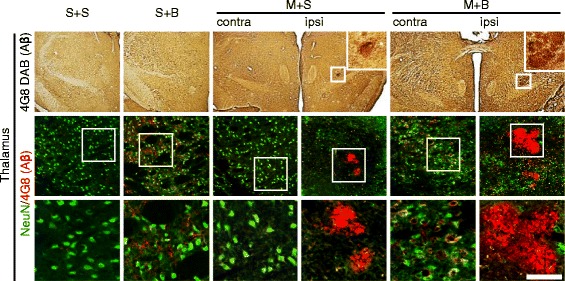
Amyloid deposit in the thalamus. Amyloid plaques are indicated in 4G8 DAB staining (insets in the first column) and double immunofluorescent labeled by neuron (NeuN) and amyloid deposit (4G8). *n* = 12 in each group; scale bar = 100 μm; MCAO middle cerebral artery occlusion, BCCAo bilateral common carotid artery occlusion, S + S sham + sham, S + B sham + BCCAo, M + S MCAO + sham, M + B MCAO + BCCAo

Amyloid deposit was investigated in the cortex (Fig. 4a). The number and area of amyloid plaques were the highest in the ipsilateral cortex of M + B rats (*p* < 0.001). Compared with that in S + S rats, the number of amyloid plaques significantly increased in the cortex of both hemispheres in M + B rats (*p* < 0.001 in the ipsilateral cortex and *p* = 0.035 in the contralateral cortex). Moreover, this number was higher in the ipsilateral cortex of M + B rats than in the ipsilateral cortex of M + S rats (*p* = 0.023, Fig. 4b). Compared with that in S + S rats, the area of amyloid plaques significantly increased in the ipsilateral cortex of M + S rats (*p* = 0.045) and M + B rats (*p* < 0.001). In M + B rats, the area increased in the ipsilateral cortex compared to the contralateral cortex (*p* = 0.008, Fig. 4c). Densely conglomerated amyloid plaques in the ipsilateral thalamus, which were reported as secondary neurodegeneration after MCAO, were more frequently observed in M + B rats (5/12, 41.7%) than in M + S rats (2/12, 16.7%), although this result did not reach statistical significance (*p* = 0.178, Fig. 5).

### Distribution of AQP4

**Fig. 6:**
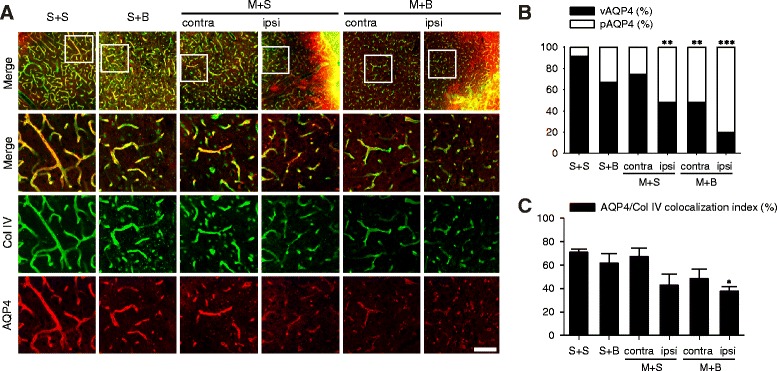
**a** Distribution patterns of AQP4 immunoreactivity. **b** Ratio of vascular and parenchymal AQP4. **c** AQP4/Col IV colocalization index. *n* = 5 to 7 in each group; scale bar = 50 μm; *p < 0.05, **p < 0.01, and ***p < 0.001 on post hoc analysis compared to S + S; vAQP4 vascular AQP4, pAQP4 parenchymal AQP4, Col IV collagen IV, MCAO middle cerebral artery occlusion, BCCAo bilateral common carotid artery occlusion, S + S sham + sham, S + B sham + BCCAo, M + S MCAO + sham, M + B MCAO + BCCAo

Along with increased neuroinflammation, AQP4 distribution changed from normal perivascular pattern to scattered parenchymal pattern (Fig. 6a). Vascular AQP4 comprising more than 90% in S + S rats decreased to 20% in the ipsilateral cortex of M + B rats, whereas the opposite effect was observed for the parenchymal AQP4 (*p* < 0.001, Fig. 6b). Compared with that in S + S rats, this pattern shift was significant in the ipsilateral cortex of M + S and both cortex of M + B rats. Colocalization of AQP4 with collagen IV decreased in the ipsilateral cortex of M + B rats followed by the ipsilateral cortex of M + S and contralateral cortex of M + B (*p* = 0.009, Fig. 6c). Compared with that in S + S rats, the AQP4 colocalization index significantly decreased in the ipsilateral cortex of M + B rats (*p* = 0.025).

### Hippocampus

**Fig. 7:**
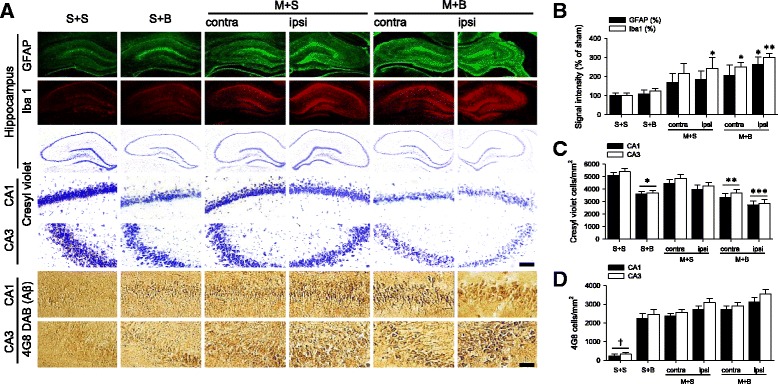
**a** Hippocampus and its magnification in CA1 and CA3 regions. Scale bar = 100 μm. **b** Neuroinflammation measured by astroglial (GFAP) and microglial (Iba1) activation ant its quantification. *n* = 7 to 8 in each group; *p < 0.05 and **p < 0.01 on post hoc analysis compared to S + S. **c** Neuronal cell death measured by cresyl violet and its quantification. *n* = 8 to 10 in each group; *p < 0.05, **p < 0.01, and ***p < 0.001 on post hoc analysis compared to S + S. **d** Amyloid-positive degenerative neurons measured by 4G8 and its quantification. *n* = 6 to 8 in each group, †*p* < 0.001 on post hoc analysis compared to other groups; MCAO middle cerebral artery occlusion, BCCAo bilateral common carotid artery occlusion, S + S sham + sham, S + B sham + BCCAo, M + S MCAO + sham, M + B MCAO + BCCAo

Neuroinflammation, neuronal cell death, and amyloid-positive degenerative neurons in the hippocampus were investigated (Fig. 7a). Neuroinflammation increased in the ipsilateral cortex of M + B rats, which was significantly higher than that of S + S rats (*p* = 0.042 in GFAP and *p* = 0.002 in Iba1, Fig. 7b). Both neuronal cell death and number of amyloid-positive degenerative neurons were significantly different among groups (*p* < 0.001 in CA1 and CA3, Fig. 7c and d, respectively). Compared to that in S + S rats, neuronal cell death was prominent in S + B (*p* < 0.05) and M + B rats (*p* < 0.01) and the number of amyloid-positive degenerative neurons was higher in all groups (*p* < 0.001).

## Discussion

Our experimental results suggest that CCH may contribute to the development of PSD by interfering with amyloid clearance through the glymphatic pathway and concomitant neuroinflammation. Therapeutic strategy alleviating these pathologies may be a promising approach to prevent PSD in patients who experienced stroke.

Although the prevalence of PSD is relatively high, reaching up to 30% of the stroke survivors as they age [2, 6], not all the patients who experienced a stroke develop PSD. Clinical factors including aging, low education level, pre-stroke cognitive decline, hypertension, diabetes, and atrial fibrillation or neuroimaging findings including silent infarcts, global cerebral atrophy, medial temporal lobe atrophy, and white matter changes are suggested as risk factors or predictors of the development of PSD [6]. Long-standing exposure to vascular risk factors such as hypertension, diabetes, or atrial fibrillation can deteriorate cerebral microperfusion [23]. Neuroimaging findings associated with cognitive impairments such as silent infarcts, global cerebral atrophy, and white matter changes can be consequences of CCH [24, 25]. We hypothesized that underlying CCH may be one of the main predisposing factors for the development of PSD among stroke survivors.

To test this hypothesis, we used a unique experimental design that can show how CCH contributes to the development of PSD after stroke. Most of the previous experimental studies on post-stroke cognitive impairment were performed using animal stroke models such as MCAO [26, 27]. However, these animal models may be insufficient to reflect complex clinical settings for the development of PSD that occurs more frequently when underlying CCH was combined. In our study, M + B rats mimicking a clinical setting where CCH is superimposed after stroke may be more appropriate to study complex interactions between CCH and territorial infarction for the development of PSD than a simple stroke model. M + B rats showed the worst performance in water maze task compared to other groups, whereas the performance of M + S rats was not impaired compared to that of S + S rats. In other words, performance based on spatial memory was synergistically impaired when BCCAo was superimposed on MCAO. These experimental findings suggest that cognitive impairment can be significantly aggravated when CCH is combined with territorial infarcts. Predisposing conditions such as CCH can be more important in PSD development than stroke itself.

The infarct volume was not different between M + S and M + B rats. Although the recovery of the motor function measured by mNSS was delayed in M + B rats, the swimming speed during the water maze task was not different among groups. The worst performance of M + B rats in water maze task may be the consequence of a post-stroke neurodegenerative process rather than larger infarct volume or impaired motor function. These findings appear to be consistent with clinical findings reporting that some stroke patients develop PSD and some do not, although they had similar infarct volume. In addition, PSD can develop even in stroke survivors with complete motor recovery. Hence, we asked which pathology could be a culprit to trigger or aggravate a post-stroke neurodegenerative process in the development of PSD other than infarct volume itself.

Here, we showed that amyloid deposit along with neuroinflammation was significantly enhanced in the ipsilateral cortex of M + B rats compared to that in M + S rats. CCH induced by BCCAo may have enhanced neuroinflammation and amyloid pathology leading to the neurodegenerative process in the ipsilateral cortex of stroke, especially in the peri-infarct area. Interestingly, amyloid deposit in M + B rats was enhanced not only in the ipsilateral cortex but also in the contralateral cortex and ipsilateral thalamus. In a rat model of transient MCAO, stress proteins such as heat shock protein were reported to be inducible not only in the peri-infarct area but also in remote contralateral cortex areas beyond the vascular territory through the mechanism of spreading depression [28]. Amyloid deposit in the ipsilateral thalamus after MCAO in rats was reported as a secondary neurodegeneration with amyloid pathology [7, 29–31]. In our study, this phenomenon was strongly enhanced in M + B rats compared to that in M + S rats, suggesting that CCH may also work as an aggravating factor for the secondary neurodegeneration in the ipsilateral thalamus after stroke. In our study, neuronal cell death and amyloid-positive degenerative neurons along with neuroinflammation in the hippocampus increased when CCH was superimposed on the stroke. Although the underlying mechanism needs further investigation, the effect of CCH on amyloid deposit after stroke may be more diffuse than expected, involving whole brain hemispheres beyond the peri-infarct area.

Post-stroke neurodegenerative processes including amyloid pathology may be more dynamic than expected. Rapid increase of amyloid plaques in the peri-infarct area within a week after the experimental stroke and subsequent clearance over the next few weeks were reported in transgenic mouse models of amyloid deposit [32, 33]. In the transient MCAO rat model, amyloid precursor protein and amyloid staining increased in the peri-infarct area for 1 week after stroke and faded over the following months [7–10]. Amyloid deposit in the peri-infarct area increased more in older than in younger rats, suggesting that the aged brain is more susceptible to the amyloid deposit after stroke [10]. A human study using amyloid positron-emission tomographic (PET) scan showed that amyloid deposit was higher in the ipsilateral peri-infarct area in patients with recent infarcts [34]. However, a further study performed by same authors showed no significant increase of amyloid deposit in the peri-infarct area [35]. Although further studies are needed to explain these conflicting results, the dynamic nature of amyloid deposit and clearance suggested by experimental animal studies may explain this discrepancy. Even though amyloid deposit occurred after stroke in a human brain, if it was cleared rapidly, amyloid PET scan could not have detected any increase of amyloid deposit. If amyloid PET scan could be performed in selected patients who have underlying pathologies such as AD, VD, or white matter lesions that are well-known risk factors for PSD, results of amyloid PET scan could be positive in these patients who are more susceptible to amyloid deposit after stroke. This hypothesis appears consistent with the clinical finding that stroke patients with underlying amyloid deposit showed a more severe and rapid cognitive decline over 3 years compared to those without underlying amyloid deposit [36].

Next, we questioned how CCH accelerates amyloid deposit in the peri-infarct area. Among various mechanisms mediating amyloid clearance, we hypothesized that ISF bulk flow clearance rather than other enzymatic degradation clearance may be more associated with vascular factors such as CCH. Glymphatic pathway has been reported as a brain interstitial metabolic solute clearance system comprised of a para-arterial CSF influx, a para-venous ISF clearance, and a trans-parenchymal pathway that is dependent on astroglial water transport via AQP4 water channel [14–17]. Metabolic waste proteins such as amyloid, tau, and synuclein can be targets for the clearance of glymphatic pathway and its impairments can lead to pathologic accumulation in the brain leading to neurodegenerative diseases such as AD, Parkinson’s disease, and traumatic brain encephalopathy [14–17]. Cerebral arterial pulsation has been suggested as a possible driving force for glymphatic pathway maintaining ISF bulk flow [14, 37]. In our study, a permanent ligation of both common carotid arteries (i.e. BCCAo surgery) may have attenuated arterial pulsation in cerebral arteries. Decreased ISF bulk flow may have hindered interstitial metabolic solute clearance through the glymphatic pathway. Consequently, perturbed amyloid clearance through the glymphatic pathway may have been attributed to the amyloid deposit in the peri-infarct area in M + B rats. Hence, we investigated the distribution of AQP4 water channels, which play a key role for the astroglial water transport in the glymphatic pathway. Perivascular distribution of AQP4 in S + S rats, implicating normal functioning water channel in the glymphatic pathway, changed to scattered parenchymal pattern in M + B rats. Moreover, colocalization of AQP4 over vascular collagen IV signal decreased. Dislocation of AQP4 water channels from perivascular expression on the end-feet of astrocytes to scattered parenchymal patterns may reflect dysfunction of glymphatic pathway. Taken together, perturbed glymphatic pathway suggested by the aberrant dislocation of AQP4 water channel along with neuroinflammation may have contributed to the post-stroke amyloid deposit in M + B rats. However, detailed mechanisms of PSD in our study need further investigation. First, the dynamics of amyloid deposit after stroke over time remain to be addressed. Second, the mechanism of how CCH affects the functional and structural changes of glymphatic pathway related to the amyloid clearance warrants further study. Third, further experiments using aged animals with comorbidities such as atherosclerosis, hypertension, diabetes, obesity, or metabolic syndrome are needed to investigate neurodegeneration which occurs mostly in elderly patients with these chronic disease conditions [38–40]. Mechanism focused on the interaction between neuroinflammation and cerebral perfusion deficits in patients with PSD also warrants further study [41].

## Conclusions

Our proof-of-concept study provides experimental evidence for the mechanism of PSD, suggesting that CCH may contribute to the development of PSD by interfering with amyloid clearance after stroke through the glymphatic pathway and concomitant neuroinflammation. Therapeutic strategies to improve brain metabolic waste clearance through glymphatic pathway may be promising approaches to prevent PSD after stroke.

## Abbreviations

4G8: Amyloid beta_17-24_
AD: Alzheimer’s disease
AQP4: Aquaporin 4
BCCAo: Occlusion of bilateral common carotid arteries
CCH: Chronic cerebral hypoperfusion
CSF: Cerebrospinal fluid
DAB: Diaminobenzidine
GFAP: Glial fibrillary acidic protein
Iba-1: Ionized calcium-binding adapter molecule-1
ISF: Interstitial fluid
MCAO: Occlusion of the middle cerebral artery
mNSS: Modified neurologic severity scores
PBS: Phosphate-buffered saline
PET: Positron-emission tomographic
PFA: Paraformaldehyde
PSD: Post-stroke dementia
ROI: Region of interest
VD: Vascular dementia

## References

[1] Murray CJ, Lopez AD (1997). Global mortality, disability, and the contribution of risk factors: Global Burden of Disease Study. Lancet.

[2] Ivan CS, Seshadri S, Beiser A, Au R, Kase CS, Kelly-Hayes M (2004). Dementia after stroke: the Framingham study. Stroke.

[3] Censori B, Manara O, Agostinis C, Camerlingo M, Casto L, Galavotti B (1996). Dementia after first stroke. Stroke.

[4] O'Brien JT, Thomas A (2015). Vascular dementia. Lancet.

[5] Langa KM, Foster NL, Larson EB (2004). Mixed dementia: emerging concepts and therapeutic implications. JAMA.

[6] Leys D, Henon H, Mackowiak-Cordoliani MA, Pasquier F (2005). Poststroke dementia. Lancet Neurol.

[7] van Groen T, Puurunen K, Maki HM, Sivenius J, Jolkkonen J (2005). Transformation of diffuse beta-amyloid precursor protein and beta-amyloid deposits to plaques in the thalamus after transient occlusion of the middle cerebral artery in rats. Stroke.

[8] Hiltunen M, Makinen P, Peraniemi S, Sivenius J, van Groen T, Soininen H (2009). Focal cerebral ischemia in rats alters APP processing and expression of Abeta peptide degrading enzymes in the thalamus. Neurobiol Dis.

[9] Nihashi T, Inao S, Kajita Y, Kawai T, Sugimoto T, Niwa M (2001). Expression and distribution of beta amyloid precursor protein and beta amyloid peptide in reactive astrocytes after transient middle cerebral artery occlusion. Acta Neurochir.

[10] Popa-Wagner A, Schroder E, Walker LC, Kessler C (1998). Beta-amyloid precursor protein and ss-amyloid peptide immunoreactivity in the rat brain after middle cerebral artery occlusion: effect of age. Stroke.

[11] Pasquier F, Leys D (1997). Why are stroke patients prone to develop dementia?. J Neurol.

[12] Hennerici MG (2009). What are the mechanisms for post-stroke dementia?. Lancet Neurol.

[13] Zlokovic BV (2011). Neurovascular pathways to neurodegeneration in Alzheimer’s disease and other disorders. Nat Rev Neurosci.

[14] Tarasoff-Conway JM, Carare RO, Osorio RS, Glodzik L, Butler T, Fieremans E (2015). Clearance systems in the brain-implications for Alzheimer disease. Nat Rev Neurol.

[15] Kress BT, Iliff JJ, Xia M, Wang M, Wei HS, Zeppenfeld D (2014). Impairment of paravascular clearance pathways in the aging brain. Ann Neurol.

[16] Iliff JJ, Nedergaard M (2013). Is there a cerebral lymphatic system?. Stroke.

[17] Bakker EN, Bacskai BJ, Arbel-Ornath M, Aldea R, Bedussi B, Morris AW (2016). Lymphatic clearance of the brain: perivascular, paravascular and significance for neurodegenerative diseases. Cell Mol Neurobiol.

[18] Choi BR, Kim DH, Back DB, Kang CH, Moon WJ, Han JS (2016). Characterization of white matter injury in a rat model of chronic cerebral hypoperfusion. Stroke.

[19] Choi BR, Kwon KJ, Park SH, Jeon WK, Han SH, Kim HY (2011). Alternations of septal-hippocampal system in the adult Wistar rat with spatial memory impairments induced by chronic cerebral hypoperfusion. Exp Neurobiol.

[20] Choi BR, Lee SR, Han JS, Woo SK, Kim KM, Choi DH (2011). Synergistic memory impairment through the interaction of chronic cerebral hypoperfusion and amlyloid toxicity in a rat model. Stroke.

[21] Kim HY, Singhal AB, Lo EH (2005). Normobaric hyperoxia extends the reperfusion window in focal cerebral ischemia. Ann Neurol.

[22] Li Y, Chen J, Wang L, Lu M, Chopp M (2001). Treatment of stroke in rat with intracarotid administration of marrow stromal cells. Neurology.

[23] Skoog I (2000). Vascular aspects in Alzheimer’s disease. J Neural Transm Suppl.

[24] Norrving B (2015). Evolving concept of small vessel disease through advanced brain imaging. J Stroke.

[25] Kim BJ, Lee SH (2015). Prognostic impact of cerebral small vessel disease on stroke outcome. J Stroke.

[26] Yonemori F, Yamada H, Yamaguchi T, Uemura A, Tamura A (1996). Spatial memory disturbance after focal cerebral ischemia in rats. J Cereb Blood Flow Metab.

[27] Andersen MB, Zimmer J, Sams-Dodd F (1999). Specific behavioral effects related to age and cerebral ischemia in rats. Pharmacol Biochem Behav.

[28] Popp A, Jaenisch N, Witte OW, Frahm C (2009). Identification of ischemic regions in a rat model of stroke. PLoS One.

[29] Zhang J, Zhang Y, Xing S, Liang Z, Zeng J (2012). Secondary neurodegeneration in remote regions after focal cerebral infarction: a new target for stroke management?. Stroke.

[30] Zhang Y, Xing S, Zhang J, Li J, Li C, Pei Z (2011). Reduction of beta-amyloid deposits by gamma-secretase inhibitor is associated with the attenuation of secondary damage in the ipsilateral thalamus and sensory functional improvement after focal cortical infarction in hypertensive rats. J Cereb Blood Flow Metab.

[31] Loos M, Dihne M, Block F (2003). Tumor necrosis factor-alpha expression in areas of remote degeneration following middle cerebral artery occlusion of the rat. Neuroscience.

[32] Garcia-Alloza M, Gregory J, Kuchibhotla KV, Fine S, Wei Y, Ayata C (2011). Cerebrovascular lesions induce transient beta-amyloid deposition. Brain.

[33] Van Nostrand WE, Davis J, Previti ML, Xu F (2012). Clearance of amyloid-beta protein deposits in transgenic mice following focal cerebral ischemia. Neurodegener Dis.

[34] Ly JV, Rowe CC, Villemagne VL, Zavala JA, Ma H, Sahathevan R (2012). Subacute ischemic stroke is associated with focal 11C PiB positron emission tomography retention but not with global neocortical Aβ deposition. Stroke.

[35] Sahathevan R, Linden T, Villemagne VL, Churilov L, Ly JV, Rowe C (2016). Positron emission tomographic imaging in stroke: cross-sectional and follow-up assessment of amyloid in ischemic stroke. Stroke.

[36] Liu W, Wong A, Au L, Yang J, Wang Z, Leung EY (2015). Influence of amyloid-beta on cognitive decline after stroke/transient ischemic attack: three-year longitudinal study. Stroke.

[37] Iliff JJ, Wang M, Zeppenfeld DM, Venkataraman A, Plog BA, Liao Y (2013). Cerebral arterial pulsation drives paravascular CSF-interstitial fluid exchange in the murine brain. J Neurosci.

[38] Buga AM, Ciobanu O, Badescu GM, Bogdan C, Weston R, Slevin M (2016). Up-regulation of serotonin receptor 2B mRNA and protein in the peri-infarcted area of aged rats and stroke patients. Oncotarget.

[39] Buga AM, Di Napoli M, Popa-Wagner A (2013). Preclinical models of stroke in aged animals with or without comorbidities: role of neuroinflammation. Biogerontology.

[40] Sandu RE, Buga AM, Uzoni A, Petcu EB, Popa-Wagner A (2015). Neuroinflammation and comorbidities are frequently ignored factors in CNS pathology. Neural Regen Res.

[41] Popa-Wagner A, Buga AM, Tica AA, Albu CV (2014). Perfusion deficits, inflammation and aging precipitate depressive behaviour. Biogerontology.

